# Nitrogen fertilization modifies organic transformations and coatings on soil biogeochemical interfaces through microbial polysaccharides synthesis

**DOI:** 10.1038/s41598-019-55174-y

**Published:** 2019-12-10

**Authors:** Xizhi Huang, Georg Guggenberger, Yakov Kuzyakov, Olga Shibistova, Tida Ge, Yiwei Li, Bifeng Liu, Jinshui Wu

**Affiliations:** 1 Britton Chance Center for Biomedical Photonics at Wuhan National Laboratory for Optoelectronics - Hubei Bioinformatics & Molecular Imaging Key Laboratory, Systems Biology Theme, Department of Biomedical Engineering, College of Life Science and Technology, Huazhong University of Science and Technology, Wuhan, P.R. China; 2 Key Laboratory of Agro-ecological Processes in the Subtropical Region, Institute of Subtropical Agriculture, The Chinese Academy of Sciences, Changsha, 410125 China; 3 Changsha Research Station for Agricultural and Environmental Monitoring, Institute of Subtropical Agriculture, Chinese Academy of Sciences, Hunan, 410125 China; 4 Institute of Soil Science, Leibniz Universität Hannover, 30419 Hannover, Germany; 5 VN Sukachev Institute of Forest, SB-RAS, 660036 Krasnoyarsk, Russian Federation; 6 Department of Soil Science of Temperate Ecosystems, Department of Agricultural Soil Science, University of Goettingen, Göttingen, Germany; 7 Institute of Environmental Sciences, Kazan Federal University, 420049 Kazan, Russia; 8 Agro-Technology Institute, RUDN University, Moscow, Russia

**Keywords:** Carbon cycle, Geochemistry

## Abstract

The soil-water interfaces (SWI) in soil pores are hotspots for organic matter (OM) transformation. However, due to the heterogeneous and opaque nature of soil microenvironment, direct and continuous tracing of interfacial reactions, such as OM transformations and formation of organo-mineral associations, are rare. To investigate these processes, a new soil microarray technology (SoilChips) was developed and used. Homogeneous 800-μm-diameter SoilChips were constructed by depositing a dispersed Oxisol A horizon suspension on a patterned glass. Dissolved organic matter from the original soil was added on the SoilChips to mimic SWI processes. The effects of ammonium fertilization (90 mg N kg^−1^ soil) on chemical composition of SWIs were evaluated via X-ray photoelectron spectroscopy. Over 21 days, ammonium addition increased OM coatings at SWIs and modified the OM chemical structure with more alcoholic- and carboxylic-C compared to the unfertilized control. Molecular modeling of OM composition at SWIs showed that N fertilization mainly facilitated the microbial production of glucans. We demonstrated that N availability modifies the specific OM molecular processing and its immobilization on SWIs, thereby providing a direct insight into biogeochemical transformation of OM at micro-scale.

## Introduction

Soil organic matter (SOM) is the largest active terrestrial carbon (C) pool on Earth^1^. Understanding the factors controlling SOM transformation and sequestration is crucial to mitigate the increase in greenhouse gases in the atmosphere and to preserve soil fertility and plant productivity^2^. Despite of intensive studies on SOM preservation and analysis of physical, chemical and biological processes, there is still no consensus on the mechanistic understanding of SOM stabilization^3–5^. The emerging perspective of SOM preservation emphasizes that the micro-environmental characteristics control its biogeochemical fate and turnover^6,7^.

The soil-water interfaces (SWI) in pores of the micron to millimeter range were considered as hotspots for SOM cycling, given the facts that complex processes, i.e. adsorption and dissolution of SOM and its microbial-mediated transformation, occur on SWIs^8–10^. Recently a compromising soil continuum model further proposed that SOM is a continuum of progressively decomposing organic compounds^6^. Specifically, a continuum of organic fragments can be continuously processed by the decomposer community. Modification and oxidation of these organic materials will increase solubility in water as well as the protection against further decomposition at SWIs.

Nitrogen (N) availability is one of the main environmental factors controlling SOM transformation^11,12^. N fertilizers influence microbial activity and metabolism, and in return, produce more diverse metabolites, which could be immobilized on the mineral surface as stable organic-mineral compounds^13^ or as necromass^14^. To understand the complexity of the SOM behaviors and fate, accurate characterization of these key physicochemical and biological interfacial interactions at SWI is an essential step^15,16^.

Advances in *in situ* surface characterization methods have greatly improved our understanding on the spatial arrangement and characteristics of SOM on soil particle surfaces at the microscopic scale^12,17,18^. Among them, X-ray photoelectron spectroscopy (XPS) is a powerful tool used to study the first 3–10 nm of particle surfaces^19^. This tool provides insightful information on the chemical composition of surfaces in high lateral resolution and shows a preferential accumulation of OC at the surface of mineral particles^19–21^. Further, XPS informs about processes related to the chemical composition of SOM at SWIs, such as the degree of oxidative alteration of mineral-associated SOM and the stronger retention of acidic SOM components on mineral surfaces^22,23^. These sophisticated instruments were hampered to *in situ* observation of SOM interfacial processes in spatial and temporal resolutions^1,7,24^. Due to the heterogeneity and complexity of the microenvironment, the intrinsic heterogeneity of soil matrix can also bring great uncertainty to the results. Consequently, basic questions such as how N fertilization affects the OM transformation and its association to mineral surfaces on SWIs are rarely investigated.

Microfluidic techniques provide promising tools for mimicking soil environmental processes at micro to mm scale^25,26^. Recently, a new approach using microfluidic-based microarray technology (SoilChip method) was introduced to mimic the temporal formation processes of soil biogeochemical interfaces^27^. SoilChip is based on the fabrication of homogeneous 800-μm-diameter soil microarrays containing the complex soil matrix on a glass slide. The solution containing dissolved organic matter (DOM) from the original soil is used to incubate the SoilChips for starting the mineral-organic matter-microbial interactions on the SoilChip. Microorganisms can be recovered and localized on the soil micro-interfaces during the incubation. Here, we further developed and applied this SoilChip-XPS integrated technique to investigate the dynamics of the composition of a paddy SWI during the 21-d incubation. Particularly, we illustrated the temporal modification in specific chemical compositions of SOM in SWIs induced by N fertilization, with the aim to provide process-oriented information of SOM transformation response to increase of N nutrient availability.

## Materials and Methods

### Soil preparation

An Oxisol (Quaternary red soil) was sampled from a long-term fertilization experimental site (established in 1990) located in the Taoyuan Agro-ecosystem Research Station (N28°56′, E111°26′), central Hunan Province of China^28^. The cropping system was double-cropped rice with green manure annually since 1990. Soil samples were taken from the Ap horizon (0–20 cm in depth), and sieved <2-mm, where also visible particular organic residues were removed. Selected physical and chemical properties of the soil samples were determined by standard procedures taken from Huang *et al*.^27^. Organic C and total N content were 15 mg kg^−1^ and 1.3 mg kg^−1^, respectively, and the resulting C/N ratio was 11.5. The pH of soil in water was 5.1, and the concentration of dissolved organic carbon (DOC) was 30 mg C kg^−1^ soil. Composition of clay particles (<2 µm) in the Oxisol was determined using X-ray diffusion spectroscopy. Vermiculite, illite and kaolinite accounted for 16%, 37% and 47%, respectively. The CEC was 8.7 cmol kg^−1^; the active Fe measured by dithionite-citrate-bicarbonate extraction methods was 13.3 g kg^−1^.

### SoilChip experimental setup

**Figure 1:**
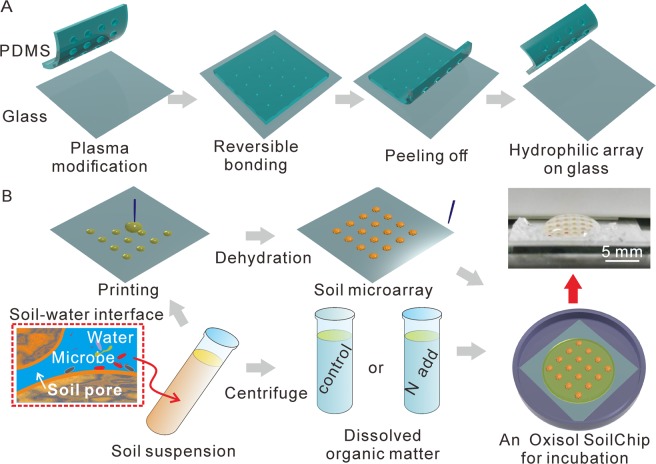
The setup of the SoilChip for mimicking the processes at soil-water interfaces. (**A**) Fabrication of the hydrophilic microarray by the patterned microfluidic technique. A polydimethylsiloxane (PDMS) stamp and glass were treated by the oxygen plasma and then stuck together. Afterwards, the PDMS stamp was peeled off. A glass chip with patterned microarray was produced. (**B**) Preparation of the SoilChip. The soil suspension was printed on the hydrophilic microarray, then self-assembled in soil microarrays. Afterwards, the soil microarray was incubated by the solution extracted from the original soil suspension under saturated moisture conditions. A physical picture of one SoilChip was demonstrated.

Homogeneous 800-μm-diameter soil microarrays on the glass chip were fabricated as reported by Huang *et al*.^27^. Briefly, the soil was pre-incubated at 25 °C and at saturated moisture in the dark for one week to recover microbial activity. Then, 1 g dry weight equivalent fresh soil was dispersed to suspension with 3 ml deionized water. The soil suspension was sieved <0.25 mm to remove particular organic residues for soil microarrays assembling use. For fabricating the homogeneous soil microarray on the glass chip, a patterned modification of the glass was applied (Fig. 1). Firstly, a polydimethylsiloxane (PDMS) molecular stamp with 800 µm diameter micro-well was produced by the standard soft lithography. Both the PDMS stamp and glass were modified by the plasma treatments, and then stuck together quickly. A few seconds later, the PDMS stamp was peeled off, and nano-scale PDMS film in the contacting area still remained on the glass^29^. Since the PDMS is hydrophobic, the patterned area with PDMS becomes hydrophobic, while the area without the PDMS remains hydrophilic (Fig. 1A). Subsequently, the soil suspension was dropped onto the 800 µm hydrophilic areas. To strengthen the structure stability of the soil microarrays on the chip, less than 0.2% (mass ratio to soil mass) polyvinyl alcohol (PVA) was added in the soil suspension as an organic cement, which has no significant effect on soil microbial activity^30^ and overall composition characteristics of soil microarrays^27^. The volume of the suspension dropped on each hydrophilic area was nearly 0.1 µl, which was equivalent to 0.3 mg of soil. The amount of soil deposited on each hydrophilic area can be controlled by the number of times of soil suspension printing. After dehydration, the soil microarrays with homogeneous surface were reassembled. To mimic the soil microenvironment, a DOM solution extracted from the pre-incubated soil was used for cultivating the soil microarrays. The DOM was extracted for 10 minutes in a 1:2 (mass:mass) soil/deionized water suspension and subsequently separated by centrifugation (8800 *g*) and pressure filtration through 0.25 µm polysulfone membrane filters (Whatman, UK). Then, the SoilChip setups were put into an incubation container with saturated air moisture and stable temperature (25 °C) (Fig. 1B). With this microarray technique it was possible to investigate the temporal behavior of SWI processes on identical samples during incubation.

In the present study, more than 30 pieces of SoilChips with soil microarrays (6 × 6) on each glass were produced, and 0.15 ml DOM solution was loaded on each chip for cultivating the microarrays (Fig. 1B). For the N addition treatment, a soil solution containing 30.6 mg DOC L^−1^ and 90 mg N L^−1^ ammonium sulfate was added onto the SoilChips. The incubated SoilChips were sampled after 0, 2, 5, 13 and 21 days of incubation. During the destructive sampling, the solution on the glass was removed by vigorous shaking by hand; the soil microarrays on the SoilChips were air-dried and characterized without further pretreatments. For each time of surfaces characterization, three SoilChips replicates were collected and only one soil dot on each SoilChip was destructively measured.

### Surface characterization

The characterization of the soil surface on the SoilChips was performed using XPS (AXIS-ULTRA DLD-600W, Manchester, UK). XPS is a sensitive surface characterizing technique with penetrating depth of 3~7 nm, which has been proved to be suitable for soil interface studies^20^. Contrary to the traditional double-sided adhesive tape methods for XPS measurements, the soil surfaces on the SoilChips were directly detected without any other preparations. The detailed measurement principle of XPS was reported by Huang *et al*.^27^. Briefly, a 300 µm-diameter area of each soil dot on the SoilChip was randomly chosen and analyzed by XPS. Sample charging during analysis was corrected based on the maximum principal C 1 s sub-peak centered at 284.8 eV. For each measurement, the wide-scan spectra covered a binding energy range from 0 to 1250 eV with a 0.4 eV step. The quantitative analysis of mass and molar ratios of Si, Al, Fe, C, O, N, Na, and Mg were calculated from the areas of the Si 2 s, Al 2p, Fe 2p, C 1 s, O 1 s, N 1 s, Na (KLL Auger), and Mg (KLL Auger) peaks from the wide-scan spectra, respectively. The detailed chemical binding information of the C and N was obtained from the high-resolution mode of C 1 s and N 1 s with a 0.05 eV step. The species of C and N was fitted by the free XPSPeak 4.0 programmer. A fixed mixed product with 20% Lorentzian/80% Gaussian shape was used for fitting the C 1 s and N 1 s peak, and the full-width-at-half-maximum (FWHM) of fitted sub-peaks was allowed to vary between 1 and 1.7^23^.

XPS can directly provide the information on the elemental composition and the chemical binding forms of C and N, but the information at the molecular level (proteins, polysaccharides, and lipids, etc.) is not given^31^. To estimate the proportion of different molecular compounds, a computation method for molecular compounds was proposed using the following elemental content ratios and the species fractions^31,32^:

1 $${({\rm{N}}/{\rm{C}})}_{{\rm{Observed}}}=0.27\,{{\rm{C}}}_{{\rm{proteins}}};$$

2 $${({\rm{C}}={\rm{O}}/{\rm{C}})}_{{\rm{Observed}}}=0.28\,{{\rm{C}}}_{{\rm{proteins}}}+0.167\,{{\rm{C}}}_{{\rm{glucans}}};$$

3 $${[{\rm{C}}{\textstyle \text{-}}({\rm{O}}/{\rm{N}})/{\rm{C}}]}_{{\rm{O}}{\rm{b}}{\rm{s}}{\rm{e}}{\rm{r}}{\rm{v}}{\rm{e}}{\rm{d}}}=0.32\,{{\rm{C}}}_{{\rm{p}}{\rm{r}}{\rm{o}}{\rm{t}}{\rm{e}}{\rm{i}}{\rm{n}}{\rm{s}}}+0.833\,{{\rm{C}}}_{{\rm{g}}{\rm{l}}{\rm{u}}{\rm{c}}{\rm{a}}{\rm{n}}{\rm{s}}};$$

4 $${[{\rm{C}}{\textstyle \text{-}}({\rm{C}}/{\rm{H}})/{\rm{C}}]}_{{\rm{O}}{\rm{b}}{\rm{s}}{\rm{e}}{\rm{r}}{\rm{v}}{\rm{e}}{\rm{d}}}=0.40\,{{\rm{C}}}_{{\rm{p}}{\rm{r}}{\rm{o}}{\rm{t}}{\rm{e}}{\rm{i}}{\rm{n}}{\rm{s}}}+1\,{{\rm{C}}}_{{\rm{l}}{\rm{i}}{\rm{p}}{\rm{i}}{\rm{d}}{\rm{s}}}.$$

(N/C)_Observed_, [C-(C/H)/C]_Observed_, [C-(O/N)/C]_Observed_, (C=O/C)_Observed_ and [C-(O/N)/C]_Observed_ are the elemental molar ratio and specific carbon species fraction relative to the C 1 s from the XPS measurements, respectively. C_proteins_, C_glucans_, and C_lipids_ are the proteins, glucans, and lipids proportions of total carbon associated with each chemical group, respectively. Such an equation system was successfully tested in bio-organic systems like bacteria cell surfaces^33^. Assuming that the SOM preserved in soil SWIs is mostly derived from microbial exudates and residues, in which protein, lipids and polysaccharides dominat^34^, these principles of the molecular compounds modeling shall be also successfully assessed and applied in the analysis of SOM on soil SWIs. In theory, the sum of protein, glucans, and lipids in microbial-derived SOM is close to 100%^31^. To assess the applicability of the computation, the sum of each fraction as the recovery rate was calculated.

### Statistical analyses

Elemental and carbon species fraction data of measurements on the SoilChips by XPS are presented as the means of three replicates with standard errors. All data were tested for normality and homogeneity of variance. We used a one-way repeated measures analysis of variance (ANOVA) to test differences between indicators at fertilized and non-fertilized SWI during the incubation time. All the analyses were tested for significance (*p* < 0.05) using the SPSS software (version 19.0; SPSS Inc., Chicago, IL, USA) for Windows.

## Results

### Effects of fertilization on dynamics of carbon and nitrogen species

**Figure 2:**
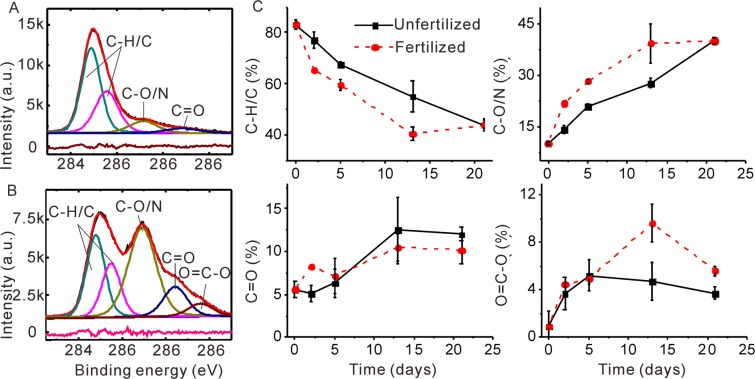
Dynamics of chemical binding species of C 1 s at the SWI of an Oxisol A horizon with or without N fertilization using X-ray photoelectric spectra. (**A**,**B**) Are representative fitting of the species from C 1 s spectra at the unfertilized SWI at the starting time and 21-d incubation, respectively. At the starting time, three main components were identified: aliphatic C, C-H/C at 284.8 and 285.5 eV, alcohol and amine C, C-O/N at 286.5 eV, and carbonyl C, C=O at 288.3 eV; after 21-d incubation, four components were identified: C-H/C at 284.8 and 285.5 eV, C-O/N at 286.5 eV, C=O at 288.3 eV, and carboxylic carbon COO- at 289.2 eV. The base line in each figure is the residual of the fitting. (**C**) Displays the temporal course of four species of carbon at the fertilized and unfertilized SWI at 21-d incubation. Data are means ± standard deviation of three measurements, except for day 2 (only one sample).

Based on the peak deconvolution, three main components of C 1 s spectra at the initially assembled SWIs were identified (Fig. 2A). While there is no indication for inorganic C, the component at a binding energy of 284.8 eV and 285.5 eV is attributed to aliphatic C (C-H/C), the peak at 286.5 eV is alcohol and amine C (C-O/N), and the component near 288.3 eV is assigned to carbonyl C (C=O)^23^. During 21-d incubation, aliphatic compounds in the unfertilized samples strongly decreased, whereas alcohol, amine, and carbonyl C increased (Fig. 2B). Further, a small peak at 289.2 eV emerged, corresponding to carboxylic C^32^. In the N fertilized samples, the proportion of alcohol C in SOM increased after 5 and 13 days, and the fraction of carboxylic C even doubled during 13 days (Fig. 2C). The main N 1 s peak on the surface before and after 21 days of incubation at 400.3 eV (Fig. S1) is attributed to protein-N^31^. After ammonium fertilization, only the protein-N peak at 400.2 eV was observed in the N1s spectra after 21-d incubation, indicating that the N was mainly assimilated and preserved as proteins at SWIs^21,35^.

### Effects of fertilization on surface composition

**Figure 3:**
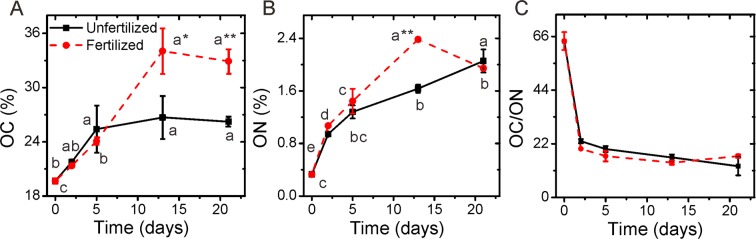
Temporal course of (**A**) C contents, (**B**) N contents, and (**C**) of the C/N ratio at soil-water interfaces (SWI) of an Oxisols A horizon with and without N addition during 21-d incubation (partial data of C and N are obtained from Huang *et al*.^27^. Data are means ± standard deviation of three measurements, except for day 2 (only one sample measured). Small letters show the significant differences between the incubation days (*p* < 0.05). The asterisks mean significance between with and without N addition treatment. One asterisk (*) means *p* < 0.05, two whiskers (**) means *p* < 0.01.

Wide-scan mode of XPS showed that after 21-d incubation, the proportion of OC and organic nitrogen (ON) contents increased at the SWIs of the non-fertilized control (Fig. 3A,B). Concurrently, the area of mineral surface represented by silicon (Si) and aluminum (Al) decreased (Fig. S2), which means that mineral surfaces were increasingly coated with SOM during the SWI formation. Nitrogen addition had a pronounced effect on the composition of the SWIs. As compared to the non-fertilized control, N fertilization led to a 25.5% larger OC content in SWIs after 21 days (increasing from 26.2% to 32.9%, Fig. 3A). Similarly, N fertilization resulted in a 46% larger ON content in SWIs after 13-d incubation. But this fertilization effect disappeared after 21 d (Fig. 3B). This is also the only sample which did not show a continuous decrease in the C/N ratio (Fig. 3C).

**Figure 4:**
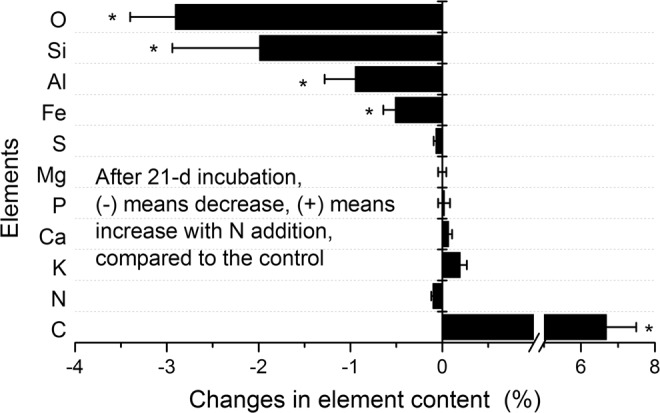
Temporal changes in main element contents between N fertilization and unfertilized control treatment during the Oxisol A horizon SWI formation within 21-d incubation. The asterisks (*) mean significance *(p* < 0.05) between with and without N addition treatment.

The contents of mineral elements, such as Si, Al and Fe, were smaller at the N amended soil as compared to the unfertilized soil (Fig. 4). The ratio of changes in the content of Si (−2%) and Al (−1%) between the fertilized and control soil was 2:1, which is close to that of the vermiculite or illite type clay minerals (2:1). This implies that the increasing organic coatings at fertilized SWIs covered clay surfaces. Along with the decrease in elements of mineral origin, the N addition induced a decrease in the relative O content at SWIs by −2.9% (Fig. 4). O content at SWIs originated from both SOM and minerals. The accumulation of OC with more oxidative state at fertilized SWIs increased content of the organic O species compared to the control soil. That means N fertilization leads SWIs to contain more carboxylic binding sites but less inorganic metal-O binding sites.

### Molecular composition modeling of organic matter

**Figure 5:**
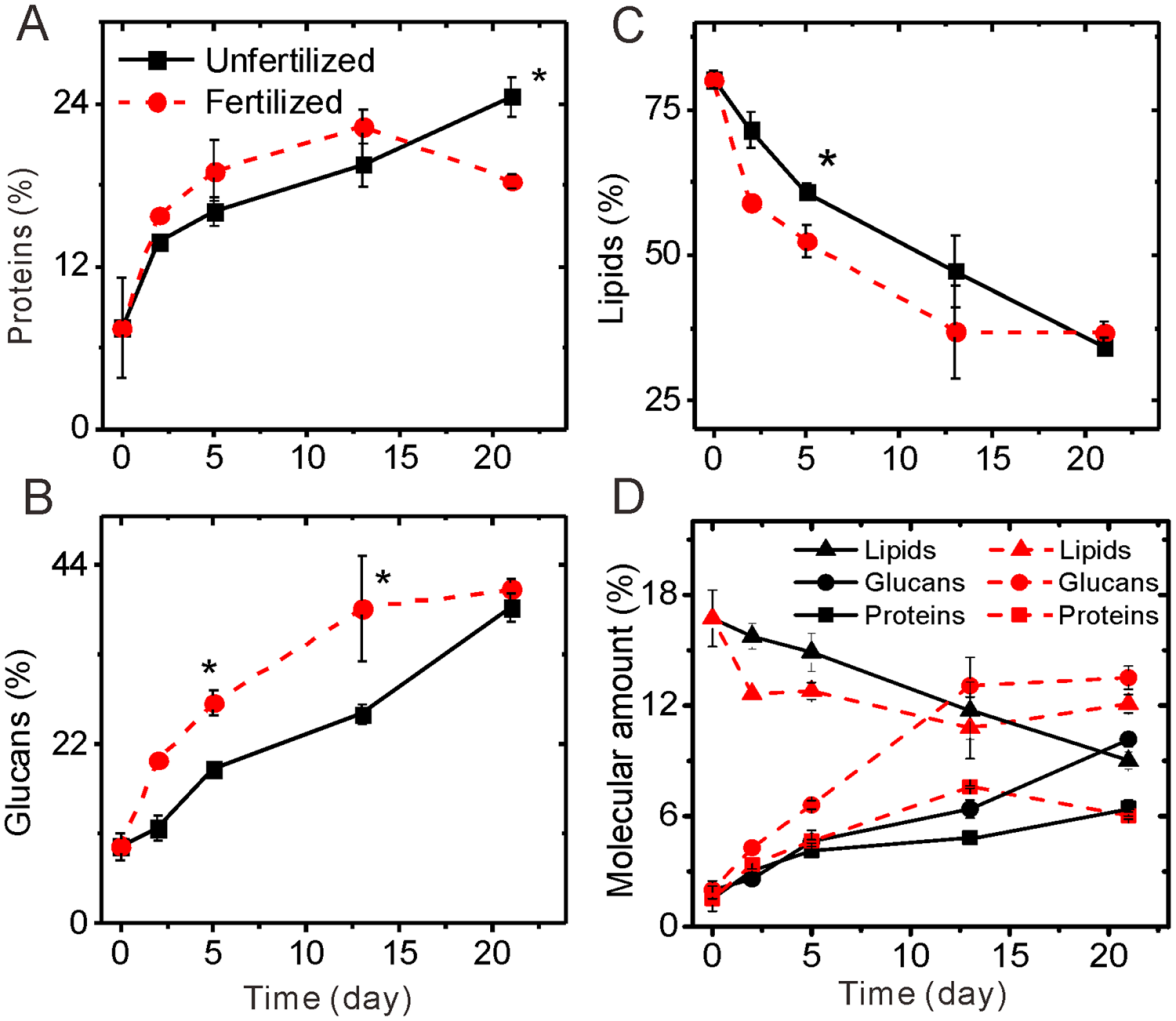
Temporal changes in the relative fraction and amount of proteins, glucans, and lipids in SOM at Oxisol A horizon SWIs during 21-d incubation.

Molecular modeling showed that N addition increased the fraction of proteins at SWI from 7.7% to 22.3% after 13 days of incubation, which was 14% higher than that of samples without N fertilization (19.6%, Fig. 5A). Compared to unfertilized controls, after 13 days of incubation the glucans were enriched by N application at the SWI (38.8% versus 25.7%, Fig. 5B). The lipid fraction decreased during the incubation, and the decrease was faster with N fertilization (Fig. 5C). However, after 21 days incubation, the protein fraction decreased to 18.8%, which was less than that of control (24.5%). A great increase in the glucans in the unfertilized control occurred from 13 to 21 days of incubation, which eliminate the effect of fertilization on the glucans on the SWI. This means that N fertilizer modified the structure of OM immobilized at SWI by its microbial processing and synthesis.

Furthermore, we calculated the amount of glucans, proteins and lipids by multiplying the total C content (Fig. 3) with the molecular fractions (Fig. 5). We found that N addition increased the relative amount of proteins after 13 days, but at day 21 the protein content between the fertilized and non-fertilized SWI was similar. Compared to unfertilized controls, the relative amount of glucans at SWIs was greatly enriched by N application at the SWI after 13 days (13.5% versus 6.4%, Fig. 5D). Such glucan accumulation by fertilization was partially offset from day 13 to day 21. The lipid fraction decreased in its proportion during the incubation, and this decrease was more pronounced with N fertilization. This is in line with more O and N enriched microbial metabolites at fertilized SWI (Fig. 2). However, contrary to the continuous decrease of lipids at non-fertilized SWI, the lipids increased at fertilized SWIs from day 13 to day 21. Such lipid enrichment with N fertilization supported our explanation for the increase in C/N ratio at the end of incubation (Fig. 3C).

## Discussion

### Suitability of the SoilChip-XPS method for studying SWI

Interfaces between liquid, solid and living (microorganisms) phases are of fundamental importance for OM biogeochemical processes along the water transport pathway through the critical zone^6,10,15,24^. However, methods for tracking the dynamics of the biogeochemical interfacial processes in soil microenvironment are still very limited due to the extreme complexity of the microenvironment. Using the SoilChip approach, the complex interfacial processes occurring in soil microenvironment can be configured for specific solutions or ingredient-specific surfaces. For example, the effects of N fertilization on the SWI properties were investigated in the present study. Despite that such microfluidic approach are artificial, it can provide novel insights into the specific evolution behavior of SWI formation in real time via sophisticated XPS measurements (Fig. 1). To the best of our knowledge, this is the first time for demonstrating the role of N fertilization on chemical properties of SWIs.

At the beginning of the experiment, higher OC and ON contents were observed on the initial SWI as compared to the bulk soil. This was also found in previous studies, where OM was concentrated more than 10-fold at the aggregate surfaces^19^. The OC content on 21-d SWI increased from initial 19.7% to 26.2% and even reached to 32.9% in the fertilized SWI (Fig. 3A). In contrast to the bulk soil analysis, SoilChip-XPS integrated technique emphasized the biological and geochemical interfacial reactions at SWI^15,27^. The OC content on SoilChip measured by XPS averages over the 300–500 µm-diameter surface area and included microbial biomass and the newly formed organo-mineral associations^27^. During the incubation, both the sorption of the DOM and also the microbial growth and deposition occurred in SWIs, thus concentrating OC^35^.

### Modeling the molecular composition of SOM from XPS data

A computation method for molecular compounds in microbial biomass was successfully established using the elemental content ratios and the species fractions of OM from the XPS measurements^31^. Microbial modification, excretion and deposition of OM at SWIs drive the development of SWI properties^36^. This fits to previous findings that most SOM is microbial-derived^34,37^. Thus, it is reasonable to apply such computation method to SWI modeling. Ramstedt *et al*. (2014) further found that such molecular modeling scheme can work well as long as there are no interfering substances, for example in the N 1 s spectra^32^. This prerequisite of the simulation is also confirmed by our high resolution N1s that sole protein N found (Fig. S1). As glucans contribute to the signal of both the C=O/C and C-(O/N)/C ratios, the calculations based on Eqs. (2) and (3), respectively, were compared. The recovery rate (sum of the protein, glucans and lipids) of computation from Eq. (3) is closer to 100% and its variability is smaller, compared to Eq. (2) (Fig. S3). Considering that alcoholic carbon is dominant in C 1s spectra compared to the carbonyl carbon, computation based on the Eq. (3) is more reasonable for molecular modeling. For all measured samples in non-fertilized and fertilized SWIs, the mean recovery rate of the molecular fractions of the computations was 96% (n = 25, Fig. S3). The residue of the recovery of the computation could originate from the small fraction of carboxylic C or phosphate-containing compounds^38^, which was not included in the equations of the computation. Despite such molecular modeling is a general proxy to only clarify three major classes of molecular components, the uniform way of data processing could provide better insight in the changing trends of OM formation in SWIs and the underlying biochemical processes.

### Effects of N addition on organic coatings in SWIs

Huang *et al*.^27^ proposed that lower carbon and nutrient availability (in terms of DOC and N contents) constrained microbial synthetic processes and contributes to a smaller SOM coating at Oxisol SWIs as compared to nutrient-rich Mollisol SWIs^27^. Investigating the same Oxisol, the present study confirms that N addition leads to faster OM synthesis and a larger C accumulation at SWIs (Fig. 3). Compared to the control condition, N fertilization facilitated more production of N-rich microbial products, which could be preferentially preserved on OM-free mineral surface as the new organo-mineral associations on the SWIs^35^. We further found that these organic coatings induced by N addition almost exclusively occurred on the vermiculite- or illite-type clay minerals (2:1), referring to the ratio of content changes in Si and Al (Fig. 4). Vermiculite mineral surface contains Si, Al, Fe and Mg. If vermiculite surface is covered by thick OM, the elements Si, Al, Fe and Mg measured by XPS simultaneously decrease. However, compared to the decrease in Si, Al and Fe, Mg content at SWIs was not consistently affected by the N fertilization (Fig. 4). Such unsynchronized changes indicated the complexity of mineral-organic interaction at the SWIs^37^. In addition to clay minerals, Fe oxides represent active surfaces for OM sorption^39^, which show high contents in of 13.3 g kg^−1^ dithionite-citrate-bicarbonate soluble Fe in the Oxisol under investigation^27^. To accurately assess whether increased organic coatings at fertilized SWIs preferentially covered on specific clay surfaces or iron oxides, mapping of organic molecular composition and mineral distribution is needed^40,41^.

Nitrogen addition modified the structure of OC accumulated at SWIs (Fig. 2). For example, after 13-d incubation N fertilization increased the fraction of alcoholic C by a factor of 1.4 and that of carboxylic C by a factor of 2 (Fig. S4). This means that N addition modified the OM structure of SWIs towards more oxidative state during the 21 days, compared to the non-fertilized controls. This is consistent with the concept of soil continuum model that N fertilization can promote microbial oxidation of SOM, with more microbial metabolic compounds including proteins or amino acids with carboxylic groups being gradually synthesized^42,43^ and preserved through the formation of organo-mineral associations on active sites of clay minerals and/or iron oxides (Fig. 4).

### Temporal dynamics of biomolecules at SWIs

OM molecular modeling showed that great differences in the biomolecules between the N fertilization and non-fertilization were observed (Fig. 5), indicating that N fertilization modified the microbial-mediated SOM transformation pathway during the SWI culture. This is also reflected by the strong linear correlation between proportion of carboxylic C and the contents of proteins, polysaccharides, and lipids in the N fertilized soil, while such a relationship was absent in non-fertilized samples (Fig. S5).

Contrary to the finding of Redmile-Gordon *et al*.^42^, who reported that under conditions of high N, polysaccharides are not produced or re-metabolized, a relative increase of polysaccharides was observed at N fertilized SWIs. This could be attributed to the special spatial and time resolved approach of the SoilChip-XPS integrated technique. The N content measured by XPS represents only several nanometers of penetrating depth. During the incubation, N availability could be gradually depleted by the microbial assimilation, and partially immobilized as peptides and/or amino acids at microbial cell surface and/or on the clay minerals at the SWIs (Figs. 2 and 3). However, the highly N enrichment found at soil SWIs with or without N addition (Fig. 2B) only reflects the condition of the outer layer of the SWI^23,31^. More N can be stored as microbial necromass or within organo-mineral associations in the sub-layer of the SWI^44^, which is not detected by XPS. Hence, microbial N uptake and sorption of ON on minerals surfaces gradually decreased the N availability during the 21-d incubation. Consequently, compared to OC, the high N availability during incubation shifted to relative lower availability, which could intrigue intensive synthesis of extracellular polymeric substances (especially polysaccharide) to overcome stress conditions^32^. Such polysaccharide synthesis process is also observed at non-fertilized SWIs at the end of incubation. Organic N immobilization strongly increased at the unfertilized SWI at the end of the incubation (Fig. 3), at the same time polysaccharides were greatly synthesized (Fig. 5).

During the incubation of SWI, the lipids decreased with or without N application. This could attribute to self-organization between the hydrophobic and hydrophilic molecules on the mineral surfaces^44^. Compared to the unfertilized control, after 13-d incubation days lipids were more depleted at N application, which is consistent with the larger contribution of the more hydrophilic glucans (Fig. 5). After 21 days, higher lipid quantities were observed in fertilized SWIs compared to the non-fertilized SWIs, which could attribute to accumulation of lipid bilayer derived from microbial cell debris^6^. Our data supports the perception that SWI is a special hotspot that microbial modification regulated the temporal synthesis dynamics of biomolecules and contributed to formation of live and distinct soil biogeochemical interfaces, which could not be detectable based on bulk soil analysis^9^.

Considering the chemical structure of polysaccharides, sugars are considered as potentially labile compounds in soils, however, polysaccharides are characterized by long turnover times, persisting not for weeks but for decades^1,45,46^. Our results directly show that instead of consumption of polysaccharides during the incubation, a highly proportion of polysaccharides in the SOM are synthesized at SWIs with or without N fertilization after 21 days (Fig. 5). Basler *et al*.^47^ showed that substrate recycling is one of the major processes explaining the long turnover time for many SOM fractions including polysaccharides^47^. We suggest that due to the gradual depletion of N bioavailability, microorganisms recycle the SOM and produce more polysaccharides, contributing to the polysaccharides preservation on SWIs.

## Conclusions

Continuous responses of the elemental and molecular properties of OM to N fertilization at Oxisol Ap hoirzon SWIs were firstly demonstrated by the SoilChip-XPS integrated technique. Compared to unfertilized soil, N fertilization modified the microbial-mediated SOM transformation during the 21-d incubation, directly confirming that SWI represent biogeochemical hotspots for OM transformation. Molecular modeling showed high proportion of polysaccharides in SWIs after 21-d incubation with and without N fertilization. This was particularly pronounced at N fertilization, which was not reported in studies based on the bulk soil analysis. Our special scope of SoilChip-XPS methodology provides complimentary *in situ* information to traditional bulk soil analysis to understand the OM biogeochemistry at soil-water interfaces. XPS is excellent for analysis of the overall chemical composition of complex surfaces, but we cannot reveal the spatiotemporal distribution of the mineral-associated SOM and microbial polysaccharides on specific minerals. Consequently, the experiments integrating the isotopic labeling with the elemental mapping techniques, such as nanoSIMS, on the SoilChip will be crucial.

## Supplementary information

supporting information

## Data Availability

All data generated or analysed during this study are included in this article.
