# Supplementary material for: Nitrogen fertilization modifies organic transformations and coatings on soil biogeochemical interfaces through microbial polysaccharides synthesis
Source: Sci Rep. 2019 Dec 10;9:18684. doi: 10.1038/s41598-019-55174-y (PMC6904481; doi:10.1038/s41598-019-55174-y)
Supplement: Supplementary file 1 — supporting information [file 41598_2019_55174_MOESM1_ESM.docx]

**Supplementary information**

Nitrogen fertilization modifies organic transformations and coatings on soil biogeochemical interfaces through microbial polysaccharides synthesis

Xizhi Huang ^a,b,c^, Georg Guggenberger ^b,d, e^, Yakov Kuzyakov ^f, g, h^, Olga Shibistova^d,e^, Tida Ge ^b,c^, Yiwei Li^a^, Bifeng Liu ^a^, Jinshui Wu ^b,c,^*

^a^ Britton Chance Center for Biomedical Photonics at Wuhan National Laboratory for Optoelectronics - Hubei Bioinformatics & Molecular Imaging Key Laboratory, Systems Biology Theme, Department of Biomedical Engineering, College of Life Science and Technology, Huazhong University of Science and Technology, Wuhan, PR China

^b^ Key Laboratory of Agro-ecological Processes in the Subtropical Region, Institute of Subtropical Agriculture, The Chinese Academy of Sciences, Changsha 410125, China

^c^ Changsha Research Station for Agricultural and Environmental Monitoring, Institute of Subtropical Agriculture, Chinese Academy of Sciences, Hunan 410125, China.

^d^ Institute of Soil Science, Leibniz Universität Hannover, 30419 Hannover, Germany.

^e^ VN Sukachev Institute of Forest, SB-RAS, 660036, Krasnoyarsk, Russian Federation

^f^ Department of Soil Science of Temperate Ecosystems, Department of Agricultural Soil Science, University of Goettingen, Göttingen, Germany

^g^ Institute of Environmental Sciences, Kazan Federal University, 420049 Kazan, Russia

^h^ Agro-Technology Institute, RUDN University, Moscow, Russia

**Corresponding Author**

*Phone: +86-731-84615224; fax: +86-731-84612685 e-mail: jswu@isa.ac.cn


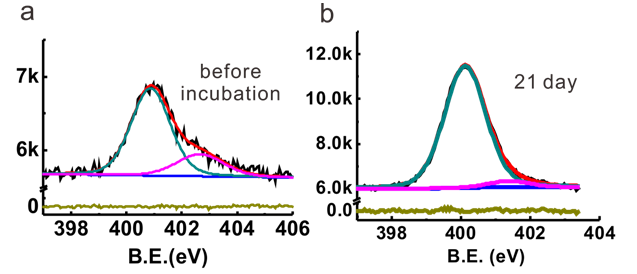


Figure S1. Spectrum of N 1s in the BGI before (a) and after (b) 21-d incubation. Mainly two species of N 1s were fitted as peptide N (400.2 eV) and primary amine N (402.3 eV). The brown line is the fitting residue of the data.


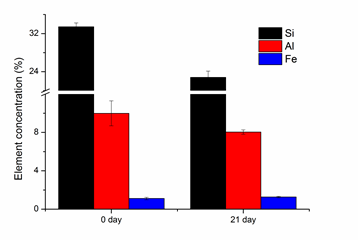


Figure S2. Example of changes in the content of Si, Al, and Fe during the BGI formation of the non-fertilized samples, formed by incubation of an Oxisol on the chip in a DOM solution extracted from the original soil. Compared to the starting point, the mineral elements (Si, Al and Fe) at the BGI decreased after 21-d incubation, attributing to coverage by the organics sorption or microbial biomass deposition.

e

**
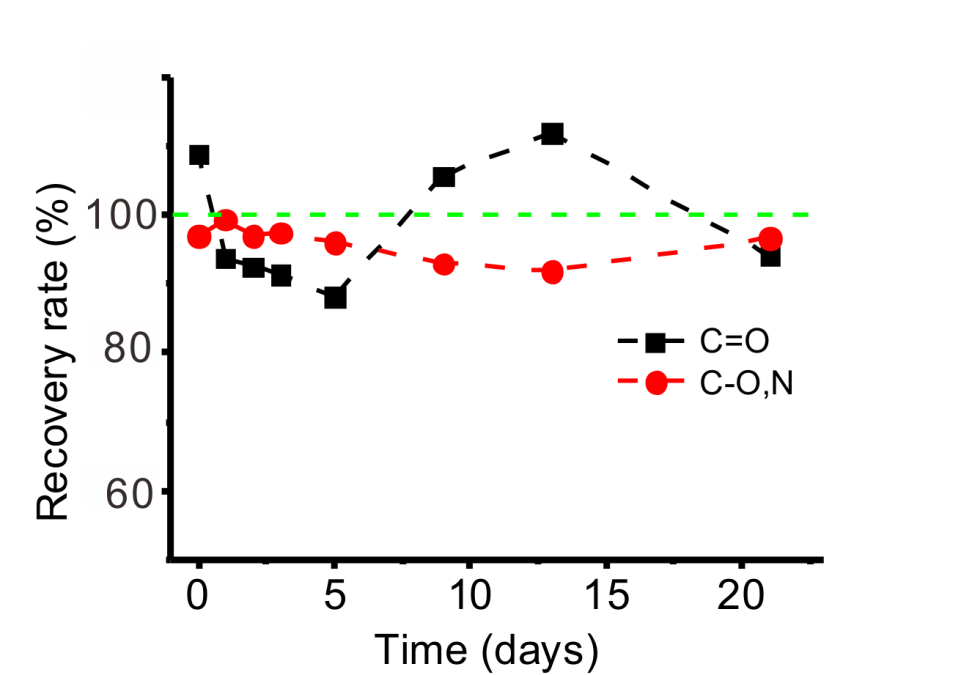
**

Figure S3 Comparison of the recovery rates of the molecular modeling during 21-d incubation based on equations (2) (C=O) and (3) (C-O,N). The results show that equation (3) is closer to 100% and its variation is smaller (red dots) than that of equation (2) (black dots)


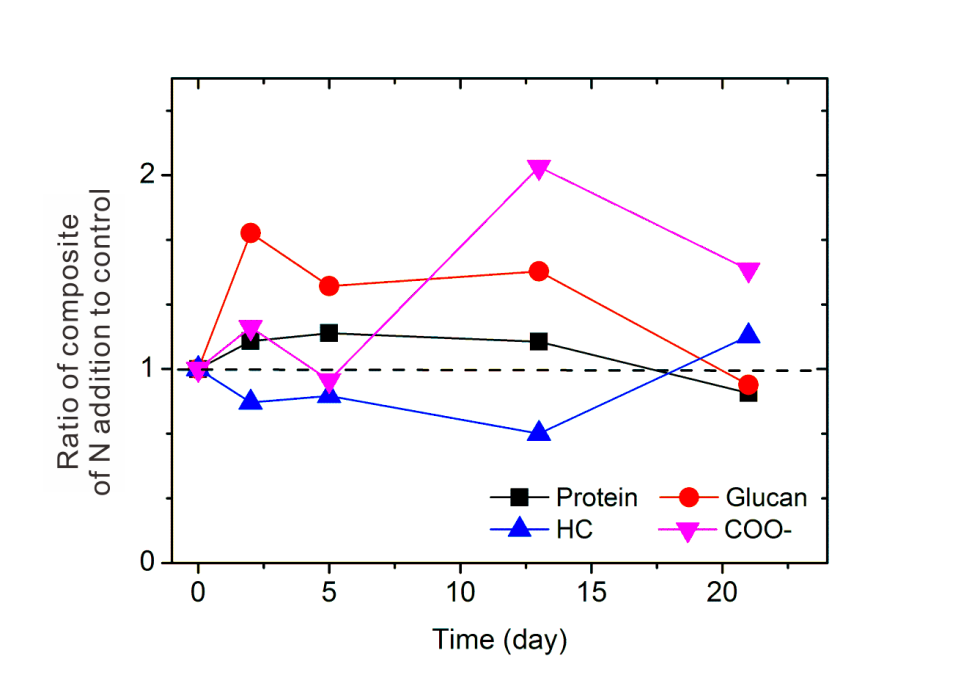


Figure S4. Ratio of biomolecules and carboxylic carbon between BGI of an Oxisol incubated for 21 d developed with N fertilization versus without N addition (control).

Figure S5. Relationship between carboxylic carbon and the biomolecules proteins, glucans, and lipids at the BGI of Oxisol without (left) and with (right) N fertilization. All presented regression lines are significant at least on the *p* < 0.05.
